# Prognostic Value of Frailty in Patients With Takotsubo Cardiomyopathy

**DOI:** 10.1002/clc.70054

**Published:** 2025-01-16

**Authors:** Carlos Diaz‐Arocutipa, Adrian V. Hernandez

**Affiliations:** ^1^ Unidad de Revisiones Sistemáticas y Meta‐análisis (URSIGET) Vicerrectorado de Investigación, Universidad San Ignacio de Loyola Lima Peru; ^2^ Health Outcomes, Policy, and Evidence Synthesis (HOPES) Group University of Connecticut School of Pharmacy Storrs Connecticut USA

**Keywords:** frailty, mortality, NIS, prognosis, Takotsubo cardiomyopathy

## Abstract

**Background:**

There is scarce data on the prognostic value of frailty in patients with Takotsubo cardiomyopathy (TCM). This study aimed to assess the association between frailty and in‐hospital outcomes in patients with TCM.

**Methods:**

Adult admissions with TCM were included using the 2016−2019 National Inpatient Sample database. The primary outcome was in‐hospital mortality and secondary outcomes included cardiogenic shock, in‐hospital cardiac arrest, stroke/transient ischemic attack (TIA), length of hospital stay, and total charges. Frailty was assessed using the hospital frailty risk score (HFRS), and admissions were divided into two groups: low risk and intermediate/high risk of frailty. Logistic regression was used to estimate odds ratios (OR) with their 95% confidence intervals (CI).

**Results:**

A total of 32 360 patients were included; the median age was 67 (58−76) years and 90% were female. The median HFRS was 2.6 (1.1−5.3). In the adjusted models, in‐hospital mortality was significantly higher in the intermediate/high risk of frailty group (OR 3.60, 95% CI 2.16−6.02) compared to the low‐risk group. Similarly, admissions with intermediate/high risk of frailty had a significantly higher risk of cardiogenic shock (OR 3.66, 95% CI 2.77−4.80), in‐hospital cardiac arrest (OR 2.57, 95% CI 1.55−4.24), and stroke/TIA (OR 5.68, 95% CI 3.51−9.20). There was a significantly higher hospital charges and length of hospital stay in the intermediate/high‐risk group. In the restricted cubic spline regression models, the frailty score was nonlinearly associated with all outcomes.

**Conclusions:**

Our results suggest that frailty is useful as a prognostic factor for in‐hospital events in patients with TCM.

## Graphical abstract

Our study aimed to assess the association between frailty and in‐hospital outcomes in patients with Takotsubo cardiomyopathy (TCM). A total of 32 360 adult admissions with TCM using the 2016−2019 National Inpatient Sample database were included. Frailty was assessed using the hospital frailty risk score (HFRS). In‐hospital mortality, cardiogenic shock, in‐hospital cardiac arrest, stroke/TIA, and the use of hospital resources were significantly higher in the intermediate/high risk compared to the low‐risk frailty group.

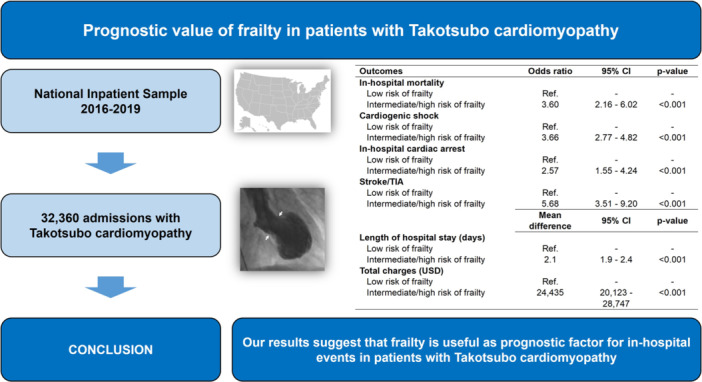

## 1 Introduction

Takotsubo cardiomyopathy (TCM) is an increasingly recognized acute cardiac condition characterized by transient left ventricular dysfunction, often mimicking acute coronary syndrome, and typically precipitated by emotional or physical stressors [1]. While initially considered a benign condition with favorable prognosis, emerging evidence suggests significant heterogeneity in outcomes among patients with TCM, with a subset experiencing adverse cardiovascular events, including thromboembolism, ventricular arrhythmias, and mortality [2]. Despite advances in understanding TCM pathophysiology and diagnosis, identifying patients at highest risk for poor outcomes remains challenging [3]. Frailty is increasingly recognized as a critical determinant of outcomes in cardiovascular diseases, as it encompasses a decline in physiological reserves and increased vulnerability to stressors, which can exacerbate the course of these conditions [4]. In patients with cardiovascular diseases, frailty has been associated with higher rates of hospitalization, adverse clinical events, and mortality. Understanding the role of frailty is particularly pertinent for TCM [4, 5], given its acute onset and the complex interplay of emotional and physical stressors involved in its pathogenesis. However, the role of frailty in predicting outcomes in TCM remains poorly understood. Therefore, this study aimed to investigate the prognostic value of frailty in patients with TCM using a nationwide database.

## 2 Methods

We performed a retrospective cohort study using the National Inpatient Sample (NIS) database for the years 2016−2019. The NIS is publicly available database administered by the Healthcare Cost and Utilization Project, containing 7 million hospital discharges each year. This database accounts for approximately 20% of all US hospitals. Adult admissions with a primary diagnosis of TCM were included, using the International Classification of Diseases, Tenth Revision (ICD–10) code I5181. Admissions with missing data for mortality and total charges were excluded. Data on sociodemographic and clinical characteristics such as age, sex, race/ethnicity, zip code‐based household income, type of admission, and comorbidities (based on Elixhauser comorbidity index) were recorded, as well as hospital characteristics. The hospital frailty risk score (HFRS) was utilized to assess each patient's frailty status [6]. This tool serves as a screening mechanism to evaluate the risk of frailty in hospitalized patients by leveraging ICD‐10 codes from administrative data. Rather than diagnosing frailty, the HFRS estimates an individual's risk of frailty through 109 comorbid diagnosis codes associated with conditions such as dementia, cognitive impairment, falls, mobility issues, and others. The scoring system assigns weights to ICD‐10 codes based on their predictive value for frailty, categorizing risk levels as low (< 5), intermediate (5−15), and high (> 15). The HFRS has demonstrated good performance in predicting adverse outcomes in hospitalized patients. In the 569‐patient validation cohort of the study that developed the HFRS score, ϰ statistics of 0.22 and 0.30 were reported when compared with the Fried and Rockwood scales, respectively [6]. In a study comparing the HFRS with the clinical frailty score in elderly hospitalized patients, the discriminative ability was found to be similar between the two scores for predicting 30‐day mortality (area under the curve [AUC] 0.80 vs. 0.80), emergency readmission (0.67 vs. 0.66), and length of hospitalization ≥ 6 days (0.73 vs. 0.68) [7]. Similarly, in another study of 5735 elderly patients hospitalized with heart failure in Australia, HFRS at admission was found to have an AUC of 0.73 for predicting 30‐day mortality [8]. Frailty was assessed as continuous data and was also stratified into the low risk of frailty (HFRS < 5) and intermediate/high risk of frailty (HFRS ≥ 5). The primary outcome was in‐hospital mortality, and the secondary outcomes were cardiogenic shock, in‐hospital cardiac arrest, stroke/transient ischemic attack (TIA), length of hospital stay, and total charges.

Categorical data were presented as absolute and relative frequencies, and continuous data as median (interquartile range [IQR] 25th percentile to 75th percentile). The chi‐square test with Rao & Scott's second‐order correction was used to compare categorical data between the frailty groups, whereas the Wilcoxon rank‐sum test was used for continuous data. For binary outcomes, logistic regression models were used to estimate crude and adjusted odds ratios (OR) with their 95% confidence intervals (CI). Liner regression models were used for continuous outcomes. Multivariable models were adjusted for age, sex, race/ethnicity, Elixhauser comorbidity index, type of admission, expected insurance payer, hospital bed size, and hospital location. In addition, restricted cubic splines were used to modeling the nonlinear association between HFRS and binary outcomes, using four knots at pre‐specified points (percentiles 5, 35, 65, and 95). In addition, we performed a subgroup analysis by sex for all outcomes. All analyses were performed using R 4.3.2 software (R Foundation for Statistical Computing, Vienna, Austria), considering a two‐tailed *p* < 0.05 as statistically significant.

## 3 Results

**Figure 1:**
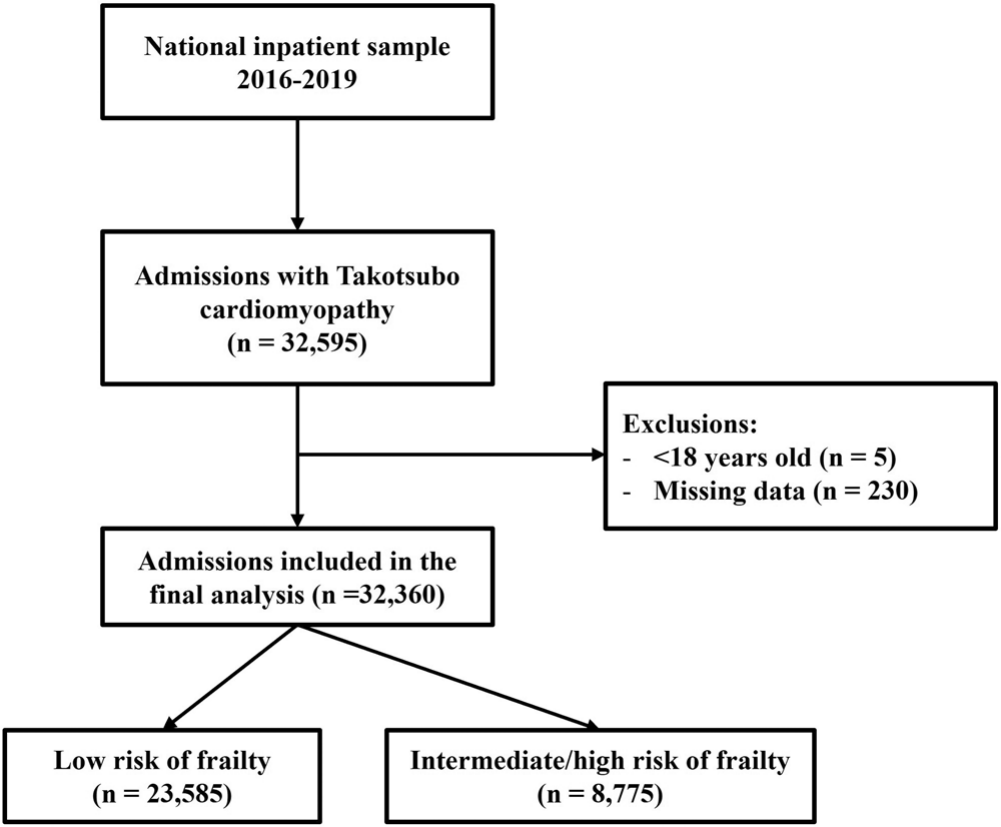
Flow diagram for the selection of study participants.

Of 32 595 patients of adults with TCM, 32 360 were included in the final analysis (Figure 1). The median age was 67 (IQR 58−76) years, 90% were female, and 80% were White (Table 1). The most common comorbidities were hypertension (65%), dyslipidemia (50%), and heart failure (37%). The median Elixhauser comorbidity index was 3 (IQR 2−5). The median length of stay was 3 (IQR 2−4) days, with most admissions occurring in large urban teaching hospitals (Table 1). The median frailty score was 2.6 (IQR 1.1−5.3), while 72.8% were at low risk of frailty and 27.2% at intermediate/high risk of frailty.

**Table 1:** Characteristics of the included admissions.

Characteristic	Overall (*n* = 32 360)	Frailty groups^b^		*p* value
		Low risk (*n* = 23 585)	Intermediate/high risk (*n* = 8775)	
Age (years)	67 (58−76)	66 (57−75)	71 (62−80)	< 0.001
Female sex	29 155 (90%)	21 400 (91%)	7755 (88%)	0.005
Race/ethnicity				0.058
White	26 010 (80%)	18 935 (80%)	7075 (81%)	
Black	2100 (6%)	1440 (6%)	660 (8%)	
Hispanic	1790 (6%)	1375 (6%)	415 (5%)	
Other	2460 (8%)	1835 (8%)	625 (7%)	
Elective admission	1425 (4%)	1045 (4%)	380 (4%)	0.869
Household income				0.400
Quartile 1	7605 (24%)	5415 (23%)	2190 (25%)	
Quartile 2	8280 (26%)	6060 (26%)	2220 (26%)	
Quartile 3	8685 (27%)	6400 (28%)	2285 (26%)	
Quartile 4	7320 (23%)	5365 (23%)	1955 (23%)	
Hypertension	21 110 (65%)	15 040 (64%)	6070 (69%)	< 0.001
Dyslipidemia	16 200 (50%)	11 820 (50%)	4380 (50%)	0.885
Congestive heart failure	12 045 (37%)	7375 (31%)	4670 (53%)	< 0.001
Chronic pulmonary disease	9345 (29%)	6070 (26%)	3275 (37%)	< 0.001
Diabetes	6500 (20%)	4265 (18%)	2235 (25%)	< 0.001
Atrial fibrillation	4535 (14%)	2755 (12%)	1780 (20%)	< 0.001
Valvular disease	4025 (12%)	2670 (11%)	1355 (15%)	< 0.001
Renal failure	3170 (10%)	1185 (5%)	1985 (23%)	< 0.001
Previous myocardial infarction	2740 (8%)	1885 (8%)	855 (10%)	0.025
Previous stroke/TIA	2495 (8%)	1355 (6%)	1140 (13%)	< 0.001
Previous PCI	1525 (5%)	1055 (4%)	470 (5%)	0.136
Previous CABG	445 (1%)	280 (1%)	165 (2%)	0.033
Elixhauser comorbidity index^a^	3 (2−5)	3 (2−4)	5 (4−6)	< 0.001
Expected insurance payer				< 0.001
Medicare	19 510 (60%)	13 105 (56%)	6405 (73%)	
Medicaid	2790 (9%)	2085 (9%)	705 (8%)	
Private	8345 (26%)	6985 (30%)	1360 (16%)	
Other	1680 (5%)	1390 (6%)	290 (3%)	
Bed size of hospital				0.906
Small	5060 (16%)	3685 (16%)	1375 (16%)	
Medium	9060 (28%)	6570 (28%)	2490 (28%)	
Large	18 240 (56%)	13 330 (57%)	4910 (56%)	
Location of hospital				0.076
Rural	1795 (6%)	1290 (5%)	505 (6%)	
Urban nonteaching	6670 (21%)	5025 (21%)	1645 (19%)	
Urban teaching	23 895 (74%)	17 270 (73%)	6625 (75%)	
Region of hospital				0.013
Northeast	6410 (20%)	4800 (20%)	1610 (18%)	
Midwest	8160 (25%)	5710 (24%)	2450 (28%)	
South	10 350 (32%)	7560 (32%)	2790 (32%)	
West	7440 (23%)	5515 (23%)	1925 (22%)	
Ownership of hospital				0.008
Government, nonfederal	2770 (9%)	1980 (8%)	790 (9%)	
Private, non‐profit	26 260 (81%)	19 010 (81%)	7250 (83%)	
Private, investor‐own	3330 (10%)	2595 (11%)	735 (8%)	
Transfer out indicator				< 0.001
Not a transfer	28 870 (89%)	22 325 (95%)	6545 (75%)	
Different acute care hospital	540 (2%)	310 (1%)	230 (3%)	
Another type of health facility	2950 (9%)	950 (4%)	2000 (23%)	
Outcomes				
In‐hospital mortality	495 (2%)	135 (1%)	360 (4%)	< 0.001
Cardiogenic shock	1615 (5%)	590 (3%)	1025 (12%)	< 0.001
In‐hospital cardiac arrest	490 (2%)	225 (1%)	265 (3%)	< 0.001
Stroke/TIA	470 (1%)	155 (1%)	315 (4%)	< 0.001
Length of hospital stay (days)	3 (2−4)	2 (2−3)	4 (3−7)	< 0.001
Total charges (USD)	37 980 (25 701−59 816)	34 460 (24 248−51 356)	52 615 (33 446−87 128)	< 0.001

*Note:* Categorical data are presented as *n* (%) and continuous data as median (25th percentile to 75th percentile).

Abbreviations: CABG, coronary artery bypass grafting; PCI, percutaneous coronary intervention; TIA, transient ischemic attack; USD, US dollars.

a The Elixhauser comorbidity index score was calculated based on 31 comorbidities.

b Risk of frailty was stratified into the low risk (hospital frailty risk score < 5) and intermediate/high risk (hospital frailty risk score ≥ 5).

**Figure 2:**
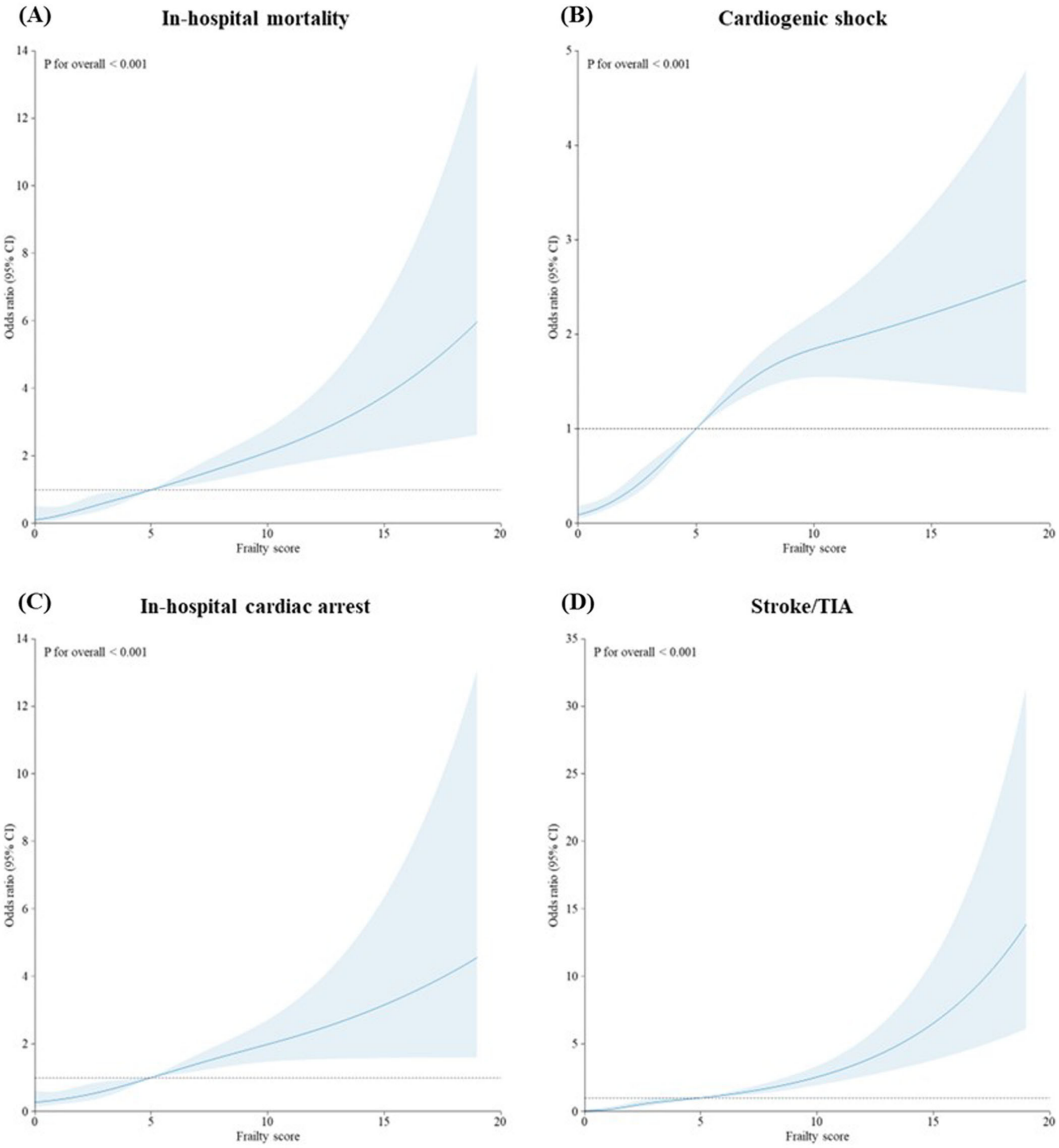
Association between hospital frailty risk score and (A) in‐hospital mortality, (B) cardiogenic shock, (C) in‐hospital cardiac arrest, and (D) stroke/TIA using a restricted cubic spline regression model. Models were adjusted for age, sex, race/ethnicity, elective admission, expected insurance payer, bed size of hospital, and location of hospital. Graphs show the odds ratios for all outcomes according to hospital frailty risk score as continuous data. The solid lines indicate the odds ratios and the gray region indicates the 95% confidence intervals. TIA, transient ischemic attack.

Overall, 2% of patients with TCM died during hospitalization. The risk of in‐hospital mortality was significantly higher in patients with intermediate/high risk of frailty (OR 3.60, 95% CI 2.16−6.02) compared to patients with low risk of frailty (Table 2). Similarly, the risk of cardiogenic shock (OR 3.66, 95% CI 2.77−4.8), in‐hospital cardiac arrest (OR 2.57, 95% CI 1.55−4.24), and stroke/TIA (OR 5.68, 95% CI 3.51−9.20) was significantly higher in the intermediate/high frailty risk group compared to the low‐risk group. Patients at intermediate/high frailty risk had significantly higher hospital costs and were hospitalized longer than those at low risk (Table 2). In the restricted cubic spline regression model, it was observed that frailty score was nonlinearly associated with all outcomes, with a gradual increase with frailty score above 5 (Figure 2). Subgroup analysis according to sex showed that, in both men and women, frailty was significantly associated with all outcomes (Table 3).

**Table 2:** Univariable and multivariable model analyses between frailty groups and outcomes.

Outcomes	Crude model			Adjusted model^a^		
	OR	95% CI	*p* value	OR	95% CI	*p* value
In‐hospital mortality						
Low risk of frailty	Ref.	—	—	Ref.	—	—
Intermediate/high risk of frailty	7.43	4.76−11.6	< 0.001	3.60	2.16−6.02	< 0.001
Cardiogenic shock						
Low risk of frailty	Ref.	—	—	Ref.	—	—
Intermediate/high risk of frailty	5.15	4.08−6.51	< 0.001	3.66	2.77−4.82	< 0.001
In‐hospital cardiac arrest						
Low risk of frailty	Ref.	—	—	Ref.	—	—
Intermediate/high risk of frailty	3.23	2.16−4.83	< 0.001	2.57	1.55−4.24	< 0.001
Stroke/TIA						
Low risk of frailty	Ref.	—	—	Ref.	—	—
Intermediate/high risk of frailty	5.63	3.65−8.68	< 0.001	5.68	3.51−9.20	< 0.001

	Mean difference	95% CI	*p* value	Mean difference	95% CI	*p* value
Length of hospital stay (days)						
Low risk of frailty	Ref.	—	—	Ref.	—	—
Intermediate/high risk of frailty	3.0	2.8−3.3	< 0.001	2.1	1.9−2.4	< 0.001
Total charges (USD)						
Low risk of frailty	Ref.	—	—	Ref.	—	—
Intermediate/high risk of frailty	33 824	29 455−38 194	< 0.001	24 435	20 123−28 747	< 0.001

Abbreviations: CI, confidence interval; OR, odds ratio; TIA, transient ischemic attack; USD, US dollars.

a Adjusted for for age, sex, race/ethnicity, elective admission, expected insurance payer, bed size of hospital, and location of hospital.

**Table 3:** Subgroup analysis between frailty groups and outcomes according to sex.

Outcomes^a^	Adjusted model^b^		
	OR	95% CI	*p* value
In‐hospital mortality			
Male	5.50	1.17–25.90	0.031
Female	3.37	1.96–5.78	< 0.001
Cardiogenic shock			
Male	12.6	5.43–29.20	< 0.001
Female	3.09	2.30–4.15	< 0.001
In‐hospital cardiac arrest			
Male	3.39	1.00–11.50	0.050
Female	2.35	1.34–4.14	0.003
Stroke/TIA			
Male	6.18	1.54–24.80	0.010
Female	5.76	3.43–9.66	< 0.001

	Mean difference	95% CI	*p* value
Length of hospital stay (days)			
Male	3.1	2.2–4.0	< 0.001
Female	2.0	1.7–2.3	< 0.001
Total charges (USD)			
Male	45 364	28 856–61 873	< 0.001
Female	21 829	17 476–26 183	< 0.001

Abbreviations: CI, confidence interval; OR, odds ratio; TIA, transient ischemic attack; USD, US dollars.

^a^The reference group is low risk of frailty (vs. intermediate/high risk of frailty) for all outcomes.

^b^Adjusted for age, race/ethnicity, elective admission, expected insurance payer, bed size of hospital, and location of hospital.

## 4 Discussion

In this large cohort of patients with TCM, we found that frailty was a strong predictor of adverse clinical outcomes, including higher rates of in‐hospital mortality, cardiogenic shock, intracardiac arrest, and stroke/TIA. In addition, frailty was associated with greater use of hospital resources.

The prognostic significance of frailty in patients with TCM likely stems from the interplay between the complex physiological alterations associated with both conditions [5]. Frailty, characterized by diminished physiological reserves and increased vulnerability to stressors [9], may exacerbate the acute physiological disturbances observed in TCM, such as catecholamine surge, myocardial stunning, and endothelial dysfunction [10]. Frail individuals may exhibit blunted compensatory mechanisms in response to these stressors, leading to a heightened susceptibility to adverse cardiovascular events [11, 12]. Furthermore, the chronic inflammatory state often associated with frailty may contribute to endothelial dysfunction and vascular damage, exacerbating the microvascular dysfunction characteristic of TCM [13, 14]. Thus, frailty may serve as a marker of the overall physiological strength in individuals with TCM, reflecting the degree of susceptibility to stressors and the potential for adverse clinical outcomes [15]. Further elucidation of the underlying pathophysiological mechanisms linking frailty and TCM prognosis is warranted to inform targeted interventions aimed at improving outcomes in this vulnerable population [15].

The increased vulnerability of frail individuals to adverse events observed in our study aligns with previous research highlighting frailty as a predictor of poor outcomes in various cardiovascular diseases [5, 16, 17]. Frailty likely exacerbates the physiological disturbances associated with TCM, predisposing patients to more severe clinical presentations and complications. Frailty assessment in patients with TCM offers clinicians an important tool for risk stratification and personalized care planning. By identifying patients with frailty early, clinicians can tailor interventions to mitigate risks associated with frailty [18], such as increased susceptibility to complications and prolonged recovery times [15, 19]. Incorporating frailty assessment into clinical practice may involve routine use of the HFRS at admission to identify patients who might benefit from enhanced monitoring and comprehensive care plans. Interventions for patients with TCM and frailty could include physical rehabilitation programs, nutritional support, and multidisciplinary management strategies aimed at addressing cognitive and mobility issues, which are common comorbidities in this population [18]. These tailored interventions can help improve patient outcomes and reduce hospital readmissions, ultimately enhancing the overall quality of care for frail individuals with TCM [20].

While our study is the first to provide information on the prognostic value of frailty in TCM, some limitations should be acknowledged. First, given the retrospective nature of the NIS database, potential measurement bias must be considered. Second, the information was extracted from administrative data based on ICD‐10 codes, thus there is a risk of miscoding of diagnoses and procedures. Third, frailty was not measured using the instruments recommended for clinical practice. While the HFRS is a validated instrument for hospitalized patients, it does not cover all geriatric and functional aspects associated with frailty, which are crucial for its full assessment. In addition, the HFRS may not capture a comprehensive picture of the overall health status of patients with TCM. Future studies should include additional assessment tools to address these multidimensional aspects of frailty. Fourth, the long‐term prognosis could not be assessed since the NIS database only reports in‐hospital events. Finally, information on laboratory, echocardiographic, and cardiac catheterization findings was not available. In addition, the NIS database does not have information on treatment received, severity of TCM or admission to the intensive care unit, therefore we do not know whether patients with frailty were treated less intensively.

## 5 Conclusions

Our results suggests that frailty has short‐term adverse prognostic implications in patients with TCM, emphasizing the importance of incorporating frailty assessment into clinical practice for risk stratification. Future large‐scale prospective studies are needed to validate our findings, as well as to develop predictive models that incorporate frailty to identify high‐risk patients at admission.

## Author Contributions

C.D‐A. involved in concept/design. C.D‐A. involved in data acquisition. C.D.‐A. and A.V.H. involved in data analysis/interpretation. C.D‐A. drafted the article. A.V.H. critically revised the article. C.D.A. and A.V.H. approved the article.

## Ethics Statement

The authors have nothing to report.

## Conflicts of Interest

The authors declare no conflicts of interest.

## Data Availability

Data are available in a public, open‐access repository. National Inpatient Sample is available online at https://hcup-us.ahrq.gov/databases.jsp.
